# Moderate thinness and its association with muscle strength of children 5–7 years in Ethiopia: a comparative cross-sectional study

**DOI:** 10.1017/S1368980025100955

**Published:** 2025-08-27

**Authors:** Melese Sinaga Teshome, Eden Alemayehu, Evi Verbecque, Sarah Mingels, Marita Granitzer, Eugene Rameckers, Teklu Gemechu Abessa, Tefera Belachew

**Affiliations:** 1 Department of Nutrition and Dietetics, Faculty of Public Health, Health Institute, Jimma University, Jimma, Ethiopia; 2 Rehabilitation Research Centre (REVAL), Rehabilitation Sciences and Physiotherapy, Hasselt University, Wetenschapspark 7, 3590 Diepenbeek, Belgium; 3 Department of Nutrition and Dietetics, Faculty of Public Health, Mizan Tepi University, Mizan, Ethiopia; 4 Musculoskeletal Research Unit, Department of Rehabilitation Sciences, Faculty of Kinesiology and Rehabilitation Sciences, Leuven University, 3000 Leuven, Belgium; 5 Research School CAPHRI, Department of Rehabilitation Medicine, Maastricht University, Maastricht, The Netherlands; 6 Centre of Expertise in Rehabilitation and Audiology, Hoensbroek, The Netherlands; 7 Department of Special Needs and Inclusive Education, Jimma University, Jimma, Ethiopia

**Keywords:** Muscle strength, Moderate thinness, Well-nourished, School-aged children, Ethiopia

## Abstract

**Objective:**

This study aimed to investigate the association between moderate thinness (MT) and muscle strength among children aged 5–7 years old in Ethiopia.

**Design:**

A school-based comparative cross-sectional study was conducted between June and July 2022. Their nutritional status (MT *v*. well-nourished (WN) was identified using BMI-for-age-and-sex; hand grip was measured using a digital grip strength dynamometer, and biceps, quadriceps and gastrocnemius strength were measured with Digital (Handheld) Dynamometry. Independent predictors of muscle strength were identified using a multivariable linear regression model.

**Setting:**

The study was conducted in Kindergarten and primary schools of Jimma Town, located in Southwest Ethiopia.

**Participants:**

Children 5–7 years old (*n* 388) with moderate thinness (MT = 194) and well-nourished peers (WN = 194).

**Results:**

Children with MT (*n* 198) had significantly lower grip strength, biceps, quadriceps and gastrocnemius muscle groups than WN children (*n* 198) (*P* < 0·001). The mean and sd of grip strength were 4·15 (sd 2·56) kg for MT and 5·6 (sd 2·04) kg for WN children. Biceps strength was 34·3 (sd 7·34) Newton (N) for MT and 48 (11·69) N for WN children. Gastrocnemius strength was 30·1 (6·9) N for MT and 45·1 (sd 9·7) N for WN children. After adjusting for background characteristics, WN children had 1·38 times higher grip strength (*β* = 1·38, *P* < 0·001), 11·22 times higher biceps strength (*β* = 11·22, *P* < 0·001), 16·70 times higher quadriceps strength (*β* = 16·70, *P* < 0·001) and 12·75 times higher gastrocnemius strength (*β* = 12·75, *P* < 0·001) than MT children.

**Conclusion:**

Children with MT had significantly lower muscle strength than their WN counterparts. This highlights the negative functional effect of wasting.

The term malnutrition refers to various disorders, varying from undernutrition, including acute or chronic undernourishment or micronutrient deficiencies, to overnutrition, including overweight and obesity^(1)^. In contrast to chronic undernutrition, acute undernutrition occurs when there is a sudden decrease in food intake and/or poor dietary quality, usually linked to diseases^(2,3)^. Forms of acute undernutrition in children may include wasting (low weight-for-height), underweight (low weight-for-age), edematous malnutrition^(4)^ and thinness (low BMI for age). Children 5 years and above with a BMI-for-age z-score between –2 and –3 are classified as having a moderate form of acute malnutrition known as moderate thinness (MT)^(5)^. Globally, 8·4 % of the girls (75 million) and 12·4 % of the boys (117 million) aged 5–19 years are categorised as thin^(6)^. A recent systematic review on school-age children (6–12 years old) in low- and middle-income countries revealed that underweight and thinness are most prevalent, ranging from 21 % to 36 % in Southeast Asian and African countries, with lower prevalence in Latin America, ranging from 6 % to 8 %^(7)^. In Ethiopia, 22 % of school-age children were found to be either wasted or thin^(8)^.

According to research by the World Bank, undernutrition is the leading risk factor for 2·9 million fatalities each year, or nearly 28 percent of all deaths in Africa^(9)^. It caused an annual loss in potential productivity estimated at 5·9 billion USD^(10)^. The risk of morbidity and mortality is higher for children with MAM who do not receive treatment, as they may advance to severe thinness^(11,12)^. Undernutrition can have long-term negative effects on economic development, especially when it occurs during childhood. According to the Cost of Hunger report in Ethiopia, the total annual cost of undernutrition in Ethiopia was estimated to be 55·5 billion ETB (1·91 billion USD), which is equivalent to 16·5 % of GDP in 2009^(13)^.

Undernutrition has a major impact on the child’s physical and cognitive functioning, thereby negatively affecting schooling, lifetime earning capacity and educational outcomes^(14–16)^ and motor performances, which are an outward sign of physical growth, malfunction in strength and power, in turn affecting perceptual-motor development^(17,18)^. It leads to this effect through alteration of body composition with reduced fat and fat-free body cell mass (i.e. muscle mass), leading to diminished physical and mental function, skeletal muscle mass and strength^(19–21)^. When dietary protein intake is below the required amount, muscles are broken down, leading to the loss of their mass, which in turn results in the deterioration of muscle strength, such as decreased grip strength^(20,22,23)^. Muscle strength is an important factor in the development of children and adolescents. It is necessary for carrying out daily activities such as self-care, walking and running, which are essential for facilitating adequate social interaction and preventing diseases in adulthood^(24)^. It is important to identify and treat undernutrition to prevent the loss of muscle mass and function, rather than just looking at body weight alone. Muscle dynamometry can be used to measure muscle strength and detect early changes in hand grip strength in children^(24–26)^.

In Ethiopia, treatment of MT is not part of the routine service except in some food-insecure areas, such as the Integrated Management of Acute Malnutrition woredas, as it is believed that the health extension programme will take care of moderately malnourished cases^(27)^. Children with MT with no access to supplementary feeding programmes experience high rates of deterioration and no improvement, as evidenced by a prospective cohort study in rural Ethiopia^(27)^. However, the relationship between muscle strength and MT has not been examined among school-age children in Ethiopia, which is crucial to evaluate and treat MT in a timely manner to prevent these consequences. This study targeted children 5–7 years old because this age is the ‘golden’ period that is developmentally sensitive when children are most active in their lives. Therefore, this study set out to investigate the association between MT and muscle strength in children aged 5–7 years old in Jimma Town, Southwest Ethiopia^(28)^.

## Methods

### Study area, period, design and participants

The manuscript, which was published earlier^(29)^, provides a detailed description.

### Sample size determination, sampling methods, procedure, data collection, measurement and definitions

**Figure 1.**
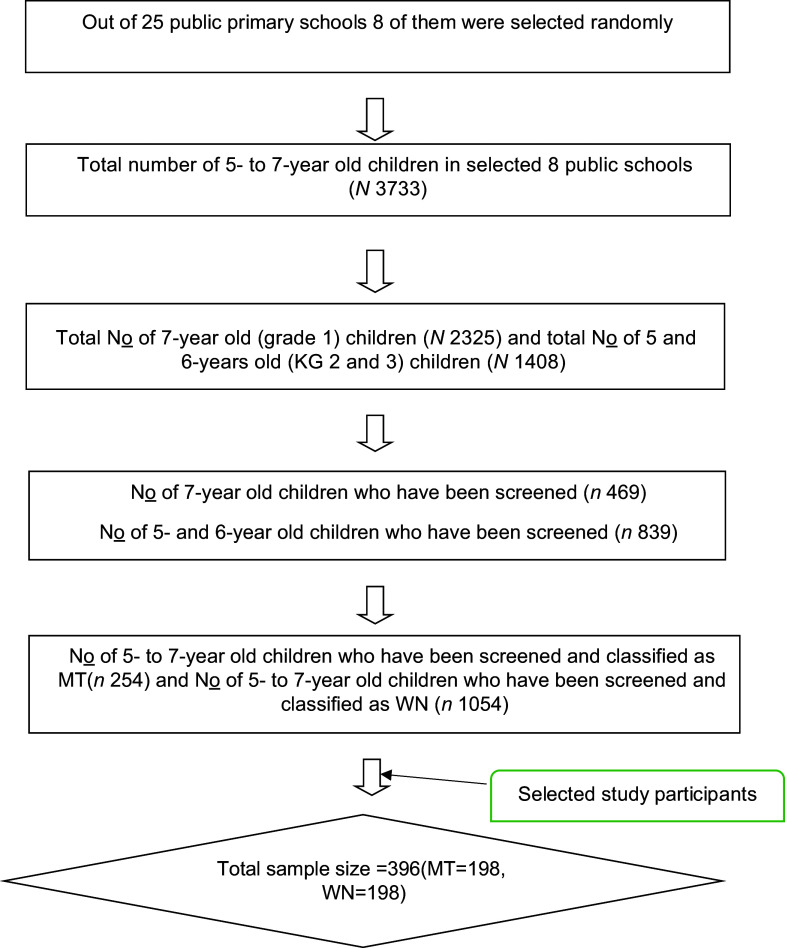
A flow chart summarising recruitment for the study.

A detailed description can be found in the manuscript that was published earlier^(29)^. The summarising recruitment procedure is shown in Figure 1.

### Anthropometric measurements

A detailed description can be found in the manuscript that was published earlier^(29)^. The height measurement was recorded to the nearest 0·1 cm^(30)^. The same assessor performed all anthropometric measurements to avoid variability. Children’s weight (kg) was measured barefoot with light clothing. The measurement was recorded to the nearest 0·1 kg^(31)^. Each mother or caregiver was asked the child’s date of birth, and if she did not remember the birth date of her child, she was probed for the approximate date of birth based on a local events calendar.

### Hand grip strength measurement

Hand grip strength (kg) was measured using a Takei Digital Grip Strength Dynamometer (Model TKK 5401, Tokyo, Japan). The dynamometer was adjusted to the participant’s hand size as required. The participants were instructed to squeeze the dynamometer with maximal force, using the preferred or dominant hand, holding it away from the body with the wrist in the neutral position and the elbow extended. The children were instructed to press the dynamometer for 3–5 s. Three tests were performed for the preferred or dominant hand. The average score was taken.

### Muscle strength of the upper limb and lower limb

Muscle strength of the lower and upper extremity muscles was measured using a digital hand-held dynamometer (HHD) model (Hoggan MicroFET2™) manual muscle tester. Before beginning the muscle strength measurement procedures, warming up for the test was performed for each child. Standard steps were followed to measure the maximum strength of the elbow flexor, knee flexor and knee extensor muscles^(32)^. The procedure taken during the Isometric strength test (HHD) measurement included: (a) Make method: the tester creates a fixed position Microfet, and the child tries to move against Microfet 2, and sitting should be adapted for each child (comfortable position); (b) three times the measurement of maximum force. When a deviation of > 20 % was observed within the three measurements, a fourth or fifth measurement was performed until the deviation was in the range of 20 %. (c) Strong verbal encouragement during the repetitions was given to produce maximum force, and three seconds of rest in between each measurement (d) With each attempt, the child gradually built up force against the HHD for about 5 s, (e) Positions for placement are standardised (Table 1), (f) lever arm is measured between the landmarks with a hard tape measure (Table 1), (g) the participant was encouraged to make the method for 5 seconds, (h) when this is the case, the measurement was stopped by saying stop and the result was recorded and (i) three consecutive measurements were done on the same arm or leg and the average value was obtained (with a 2-minutes pause between them). The test procedures for each muscle are shown in Table 1.

**Table 1.** Standard protocol hand-held dynamometry for the measurement of muscle strength of the children

Muscle group	Position	Stabilisation	Hand-held dynamometry (HHD) placement	Direction resistance- creating block
Elbow flexors	Sitting/supine Shoulder adducted elbow 90°flexed, forearm supinated, closed fist or shoulder 30°abducted, elbow 90° flexed, forearm supinated	The pelvis was stabilised using a belt or manual stabilisation	The pelvis was stabilised using a belt or manual stabilisation	Forearm distally
Quadriceps (knee extensor)	Sitting, hip and knees at 90 degrees	The pelvis stabilised in the chair using hands or belt	Anterior tibia 5 cm proximal from bimalleolar line	Block on frontal side tibia
Knee flexor	Sitting, hip and knees at 90 degrees	The pelvis stabilised in the chair using hands or belt	Anterior tibia 5 cm proximal from bimalleolar line	Block on the dorsal side of the lower leg
Gastrocnemius	Supine, knees extended, ankle in a neutral position. Foot free from the table	The pelvis was stabilised using a belt or manual stabilisation	Metatarsal heads	Block on the sole of the foot
Grip strength	Upright The elbow bent at a 90° angle	The handle is adjusted to the subject’s finger can grasp and squeeze it	with Jamar Dynamometer	subject squeezes as hard as possible and relaxes

The test started in a sitting position so that the child could familiarise themselves with the procedure and practice leg first, then arm muscles. This was followed by testing in supine and prone positions. Three attempts were made for each muscle group with the make-test technique, where resistance was gradually built up for about 5 s. Time for rest was given between trials, and measurements were varied between legs and arms to avoid fatigue. Encouragement to make the maximum effort was given in a standardised way. The maximum result for each muscle group was used. To allow as long a lever arm as possible at a location where a strong pressure on the skin did not hurt and prevented a maximal contraction, the HHD was placed distally 5 cm from the joint at the segment and tested at a place that was comfortable for the subject,^(33)^ and the protocol for testing muscle groups in children 5–15 years of age was adopted from Eek *et al.*, (2006). The position of the HHD head was marked on the skin, as was the position of the centre of the HHD head. The torque was calculated in Newton. The procedure was performed for 15–20 min.

In different studies, maximal isometric muscle strength values obtained by HHD showed that total muscle strength can be explained by using only two variables (elbow extensor muscles and knee extensor muscles), which had an R-value of 0·957, suggesting that 95·7 % of total strength variability might be explained by the strength of these two muscles^(34)^. So, for this study, those muscles and the gastrocnemius muscles were measured with HHD^(35,36)^.

### Household food insecurity

Household food insecurity was measured using the Household Food Insecurity Access Scale, which was developed by the Food and Nutrition Technical Assistance (FANTA) project and validated in Ethiopia^(37)^. For the Household Food Insecurity Access Scale measurement, each of the questions was asked with a recall period of four weeks (30 d)^(38)^. Then, food security was grouped according to the syntax in the Household Food Insecurity Access Scale^(37)^.

### Wealth index

Households are given scores based on their ownership of durable assets. After checking all the assumptions, the scores were computed using principal component analysis and ranked as poor, medium and rich.

### Statistical analysis and data quality management

A detailed description can be found in the manuscript that was published earlier^(29)^. In addition, the proper performance of each scale was checked every day by a known 1 kg standard weight before starting a measurement, and a standardisation exercise was performed during the training to capture technical errors of measurement. The technical errors of measurement were done to evaluate the interobserver and interobserver errors using a published methodology^(39)^.

## Results

### Socio-demographic characteristics of the mothers/caregivers

Out of the 396 mothers or caregivers, 388 gave complete answers, resulting in a response rate of 97·9 %. Regarding the socio-demographic characteristics, the mean standard deviation (sd) age of study mothers/caregivers was 32·08 (sd 7·57) years. The majority of participants were married, with 86·6 % of well-nourished (WN) children’s families falling into this category, while 13·9 % of MT children came from divorced families. Among the MT children, 110 mothers or caregivers, accounting for 56·7 %, were unable to read and write. More than two-thirds, 302(77·8 %) of the study participants lived in households with a family size of less than or equal to five. Additionally, 136 participants (70·1 %) came from families living in poverty among the MT children. Most participants were from food-insecure households: 185 (95·4 %) among MT children and 124 (64 %) among WN children (Table 2). These results were confirmed by the values of the technical error of measurement for weight and height in the acceptable range. For weight, intra- and inter-observer variation was 0·11 kg, which is acceptable (< 0·21 kg), whereas for height, it was 0·65 cm, which is acceptable (< 1·0 cm).

**Table 2.** Socio-demographic characteristics of mothers/caregivers of 5–7 years old with MT and WN children in Jimma town, June to July 2022

Variables	Category	Nutritional status of the child (*n* 388)					
		MT		WN		Total	
		*n* 194	%	*n* 194	%	*n* 388	%
Age (years)	≤ 19 year	7	3·6	0	0·0	7	1·8
	20–29 year	54	27·8	87	44·8	141	36·3
	30–39 year	98	50·5	88	45·4	186	47·9
	≥ 40 year	35	18·0	19	9·8	54	13·9
Ethnicity	Oromo	134	69·1	129	66·5	263	67·8
	Amhara	16	8·2	23	11·9	39	10·1
	Kafa	7	3·6	13	6·7	20	5·2
	Yem	20	10·3	11	5·7	31	8·0
	Other*	17	8·8	18	9·3	35	9·0
Religion	Muslim	103	53·1	100	51·5	203	52·3
	Orthodox	55	28·4	58	29·9	113	29·1
	Protestant	35	18·0	35	18·0	70	18·0
	Other**	1	0·5	1	0·5	2	0·5
Educational status mother/caregiver	Can’t read and write	110	56·7	39	20·1	149	38·4
	Can read and write	24	12·4	32	16·5	56	14·4
	Primary (0–8)	47	24·2	64	33·0	111	28·6
	Secondary (9–12)	11	5·7	55	28·4	66	17
	Above secondary (> 12)	2	1·0	4	2·1	6	1·5
Marital status mother/caregiver	Married and live together	109	56·2	168	86·6	277	71·4
	Married and live separately	21	10·8	5	2·6	26	6·7
	Divorced	27	13·9	14	7·2	41	10·6
	Widowed	29	14·9	6	3·1	35	9
	Single	8	4·1	1	0·5	9	2·3
Occupation mother/caregiver	Housewife	80	41·2	68	35·1	148	38·1
	Merchant	23	11·9	32	16·5	55	14·2
	Gov’t employee	6	3·1	41	21·1	47	12·1
	Private-employee	20	10·3	27	13·9	47	12·1
	Daily laborer	65	33·5	26	13·4	91	23·5
Family size	≤ 5	140	72·2	162	83·5	302	77·8
	> 5	54	27·8	32	16·5	86	22·2
Head of the household	Father	133	68·6	174	89·7	307	79·1
	Mother	61	31·4	20	10·3	81	20·9
Household food security	Secure	9	4·6	70	36·0	79	20·4
	Insecure	185	95·4	124	64·0	309	79·6
Wealth status	Poor	136	70·1	48	24·7	184	47·4
	Medium	34	17·5	39	20·1	73	18·8
	Rich	24	12·4	107	55·2	131	33·8

*Other: Silte, Dawro, and Tigre. **Other: Adventist and catholic., MT: moderate thinness, WN: well-nourished.

### Background characteristics of the children

A little over half of the children (54·6 %) were females, and among MT children, 59·8 % were females. The mean (sd) age of the children was 6·1 (sd 0·8) years. Concerning breastfeeding, 24·2 % of MT and 73·7 % of WN children were exclusively breastfed for the first 6 months of their lives. Regarding the time of initiating complementary feeding, 75·8 % of MT and 27·3 % of WN children started complementary feeding before the first 6 months of their lives, and the mean height of MT and WN children has a significant difference (Tables 3 and 4).

**Table 3.** Socio-demographic and health-related characteristics of children aged 5–7 years old with MT and WN children in Jimma town, June to July 2022

Variables	Category	Nutritional status of the child					
		MT		WN		Total	
		*n*	%	*n*	%	*n*	%
Age (years)	5 year	55	28·4	43	22·2	98	25·3
	6 year	75	38·7	87	44·8	162	41·8
	7 year	64	33·0	64	33·0	128	32·9
Sex	Male	78	40·2	98	50·5	176	45·4
	Female	116	59·8	96	49·5	212	54·6
Grade level	KG-2	67	34·5	53	27·3	120	30·9
	KG-3	81	41·8	90	46·4	171	44·1
	Grade-1	46	23·7	51	26·3	97	25·0
Walking distance from a school (time in minutes)	< 10	24	12·4	16	8·2	40	10·3
	10–14	53	27·3	30	15·5	83	21·4
	≥ 15	117	60·3	148	76·3	265	68·3
Means of transportation to come to school	Walking/foot	183	94·3	175	90·7	358	92·3
	Taxi	11	5·7	19	9·3	30	7·7
Place of delivery	Gov’t health facility	114	58·8	163	84·0	277	71·4
	Private health facility	7	3·6	18	9·3	25	6·4
	Home	73	37·6	13	6·7	86	22·2
Immunisation status of the child	Yes	136	70·1	185	95·4	321	82·7
	No	58	29·9	9	4·6	67	17·3
EBF for the 1st 6 months of life	Yes	47	24·2	143	73·7	190	49·0
	No	147	75·8	51	26·3	198	51·0
Time to start CF	Before 6 months	147	75·8	53	27·3	200	51·5
	At 6 months	36	18·6	65	33·5	101	26·0
	After 6 months	11	5·7	76	39·2	87	22·4
History of illness (within 2 weeks)	Yes	173	89·3	59	30·4	232	59·8
	No	21	10·8	135	69·6	156	40·2
Dewormed in last 6 months	Yes	44	22·7	88	45·4	132	34·0
	No	150	77·3	106	54·6	256	66·0

EBF: exclusive breastfeeding, CF: complementary feeding, MT: moderate thinness, WN: well-nourished, KG: kindergartens.

**Table 4.** Anthropometric characteristics of children aged 5–7 years old with MT and WN children in Jimma Town, June to July 2022

Anthropometric measure	Age category	Nutritional status				*P*-value
		MT		WN		
		Mean	sd	Mean	sd	
Mean height (cm)	5 year	108·4	4·5	111·3	4·85	0·003
	6 year	113·6	4·0	116·1	4·64	<0·001
	7 year	117·49	4·94	119·9	5·16	0·008
	Total	115·2	5·3	114·5	6·2	0·27
Mean weight (kg)	5 year	15·4	1·49	16·9	1·77	<0·001
	6 year	16·78	1·38	18·13	1·6	<0·001
	7 year	17·37	2·03	20·98	2·6	<0·001
	Total	16·59	1·8	18·8	2·56	<0·001

MT: moderate thinness, WN: well-nourished, total: for children in the age group of 5–7 years as a whole.

### Muscle strength of MT and WN children

**Figure 2.**
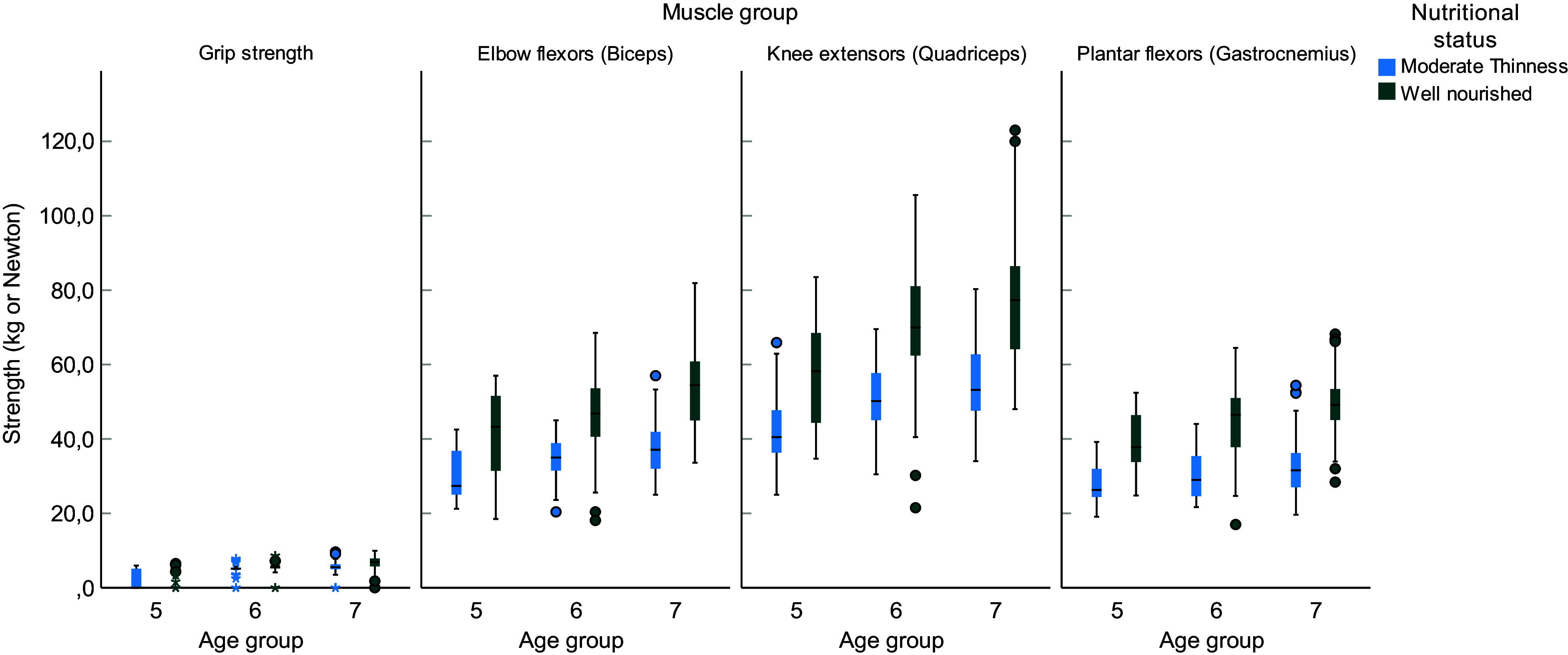
Comparisons of muscle strength among MT and well-nourished children aged 5–7 years in Jimma Town, June to July 2022.

Among the measured mean (sd) upper and lower extremity muscles and grip strength of the children between 5 and 7 years old were poorer for MT children compared with WN children (*P* < 0·05). Hand grip strength was significantly higher in 5-year-old (*P* < 0·001), 6-year-old (*P* = 0·014) and 7-year-old (*P* < 0·001) WN children, respectively, than children with MT of similar age groups. Elbow flexion of 5-year-old WN children was significantly higher compared with children with MT of the same age (*P* < 0·001). The mean handgrip strength in MT children was 4·15 kg (sd = 2·56 kg) and 5·6 kg (sd = 2·04 kg) in WN children (*P* < 0·001). Elbow flexion was 34·3 (sd = 7·34) in MT and 48 (sd = 11·69) in WN. All lower limb muscle strengths were significantly (*P* < 0·001) higher in WN children compared with MT (Table 5 and Figure 2).

**Table 5.** Independent sample *t* test for differences in muscle strength between children of age 5–7 years with MT and WN children in Jimma Town, June to July 2022

Muscle group	Age	Nutritional status						Mean difference		*P*-value
		MT			WN					
		N	Mean	sd	N	Mean	sd	Mean	95 %CI	
Grip strength (kg)	5	55	2·06	2·60	43	4·42	1·97	−2·36	−3·28, –1·44	<0·001
	6	75	4·73	1·75	87	5·40	1·68	−0·67	−1·21, –0·13	0·014
	7	64	5·26	2·28	64	6·80	1·97	−1·53	−2·28, –0·78	<0·001
	All	194	4·15	2·56	194	5·65	2·04	−1·49	−1·96, –1·03	<0·001
Elbow flexor (N)	5	55	30·03	6·41	43	41·33	10·66	−11·30	14·98, –7·62	<0·001
	6	75	35·02	5·35	87	47·13	10·59	−12·11	−14·67, –9·55	<0·001
	7	64	37·13	8·45	64	53·65	11·23	−16·51	−19·99, –13·03	<0·001
	All	194	34·30	7·34	194	48·00	11·69	−13·69	−15·64, –11·74	<0·001
Quadriceps (N)	5	55	42·90	9·15	43	57·67	14·86	−14·76	−19·91, –9·61	<0·001
	6	75	50·47	9·00	87	72·07	16·96	−21·59	−25·74, –17·45	<0·001
	7	64	53·69	11·19	64	77·72	18·55	−24·02	−29·39, –18·65	<0·001
	All	194	49·39	10·67	194	70·74	18·54	−21·35	−24·37, –18·33	<0·001
Gastrocnemius sup (N)	5	55	27·57	5·87	43	38·70	8·81	−11·12	−14·23, –8·01	<0·001
	6	75	30·02	6·09	87	45·28	9·47	−15·25	−17·70, –12·81	<0·001
	7	64	32·44	8·10	64	49·23	8·49	−16·78	−19·68, –13·87	<0·001
	All	194	30·12	6·99	194	45·12	9·76	−14·99	−16·69, –13·30	<0·001

MT: moderate thinness, WN: well-nourished, N: Newton, age 5 = 5–5·9 years; age 6 = 6–6·9 years; age 7 = 7–7·9 years.

### Independent predictors of grip strength

On multivariable linear regression, the grip strength of 5- to 7-year-old children was significantly predicted by the BMI for age, sex of the child, age of the child and birth weight of the child. WN children have 1·38 times higher grip strength than MT children (*β* = 1·38, *P* < 0·001). A unit increase in age led to an increment in grip strength by 1·41 (*β* = 1·41, *P* < 0·001), keeping other variables constant. Female children had 0·84 times lower grip strength than males (*β* = –0·84, *P* < 0·001). As the birth weight of the children increased by one gram, grip strength increased by 0·98 (*β* = 0·98, *P* = 0·001) (Table 6).

**Table 6.** Multivariable linear regression model predicting grip strength, elbow flexor, quadriceps (knee extensor) and gastrocnemius (knee flexor) muscle strength among children 5–7 years in Jimma Town, June to July 2022

Model	Grip strength			Elbow flexor muscle			Quadriceps muscle			Gastrocnemius muscle		
	*ß*	95 % CI	*P*-value	*ß*	95 % CI	*P*-value	*ß*	95 % CI	*P*-value	*ß*	95 % CI	*P*-value
BMI for age (without MT)	1·39	0·80, 1·97	< 0·001	11·22	8·64, 13·82	< 0·001	16·70	12·72, 20·69	< 0·001	12·75	10·45, 15·05	< 0·001
Sex of the child (female)	−0·84	−1·25, 0·43	< 0·001	−2·08	−3·88, 0·27	0·024	−2·77	−5·55, 0·001	0·050	−2·34	−3·95, –0·74	0·004
Age of the child (years)	1·41	1·14, 1·67	< 0·001	4·69	3·51, 5·86	< 0·001	7·21	5·39, 9·02	< 0·001	3·58	2·54, 4·63	< 0·001
Head of the house (mother)	−0·22	−0·98, 0·53	0·564	1·67	−1·67, 5·02	0·324	5·65	0·51, 10·79	0·031	−0·57	−3·54, 2·39	0·703
Birth weight of the child (gram)	0·98	0·38, 1·58	0·001	2·46	−0·17, 5·10	0·067	0·08	−3·98, 4·14	0·969	2·43	0·09, 4·78	0·042
Immunisation status of the child	0·29	−0·30, 0·89	0·336	3·03	0·41, 5·65	0·023	6·61	2·58, 10·64	0·001	3·53	1·21, 5·86	0·003
Child EBF for the first 6 months	−0·09	−0·59, 0·41	0·716	0·24	−1·97, 2·46	0·827	−1·04	−4·45, 2·36	0·547	−0·02	−1·99, 1·94	0·980
History of current illness	−0·19	−0·73, 0·34	0·478	−0·61	−2·99, 1·76	0·611	3·01	−0·65, 6·66	0·107	0·51	−1·60, 2·62	0·637
Dewormed in last 6 months	0·14	−0·33, 0·62	0·555	−0·53	−2·65, 1·58	0·619	−0·07	−3·33, 3·17	0·962	0·07	−1·80, 1·95	0·937
Food insecurity (secure)	0·37	−0·21, 0·95	0·212	−5·33	−7·89, –2·77	< 0·001	−8·32	−12·28, –4·38	< 0·001	−2·93	−5·20, –0·65	0·012
Wealth index (rich)	0·35	−0·35, 1·05	0·331	1·11	−1·24, 3·45	0·355	1·74	−1·86, 5·35	0·343	0·45	−1·63, 2·53	0·672
Maternal education (literate)	0·22	−0·31, 0·74	0·417	−3·07	−6·16, 0·03	0·052	−7·18	−11·95, –2·42	0·003	−1·39	−4·15, 1·35	0·318
Paternal education (literate)	–			1·07	−0·96, 3·10	0·301	1·36	−1·76, 4·48	0·392	0·42	−1·38, 2·22	0·647
Maximum VIF	2·582			2·582			2·571			2·571		
Adjusted R Square	33·3 %			46·2 %			47·2 %			53 %		

EBF: exclusive breastfeeding; VIF: variance inflation factor; MT: moderate thinness.

### Independent predictors of the strength of the elbow flexion

Regarding the elbow flexor muscle, BMI for age, sex of the child, age of the child, immunisation status of the child, birth weight of the child and household food insecurity were significant predictors in the multivariable linear regression model.

Well-nourished children had 11·22 times higher elbow flexor muscle strength than MT children (*β* = 11·22, *P* < 0·001). A unit increase in age led to an increment in elbow flexion of 4·69 (*β* = 4·69, *P* < 0·001), while other variables were kept constant. Female children had 2·08 times lower grip strength than males (*β* = –2·08, *P* < 0·001). Children who were immunised had 3·03 times higher elbow flexor muscles than children who were not immunised (*β* = 3·03, *P* = 0·024). Children belonging to food-secure households had 5·33 times stronger elbow flexor muscles than children in food-insecure households (*β* = –5·33, *P* < 0·001) (Table 6).

### Independent predictors of the quadriceps muscle strength

On multivariable linear regression analyses, after adjusting for other variables, BMI for age, sex of the child, age of the child, head of the house, the immunisation status of the child, maternal education and household food insecurity were significantly associated with predicting quadriceps (knee extensor) muscle strength. WN children had 16·70 times higher quadriceps (knee extensor) muscle strength than MT children (*β* = 16·70, *P* < 0·001). Maternal education being illiterate is negatively associated with quadriceps muscle strength (*β* = –7·18, *P* = 0·003). Being female decreased quadriceps muscle strength by –2·77 (*β* = –2·77, *P* = 0·05). On the other hand, being from a female-headed household (*β* = 5·65, *P* = 0·031) was positively associated with an increase in quadriceps muscle strength. For an increase in age of 1 year, quadriceps muscle strength increased by 7·2. Similarly, for children who were immunised in the quadriceps muscle, strength increased by 6·61 (Table 6).

### Independent predictors of the gastrocnemius muscle

On multivariable linear regression analyses, after adjusting for other variables, BMI for age, sex of the child, age of the child, birth weight of the child, the immunisation status of the child and household food insecurity were significantly associated with predicting gastrocnemius (knee flexor) muscle strength. WN children had 12·75 times higher gastrocnemius muscle strength than MT children (*β* = 12·75, *P* < 0·001). Being from food-secure households was negatively associated with gastrocnemius (knee flexor) muscle strength (*β* = –2·93, *P* = 0·012). Similarly, being female decreased gastrocnemius (knee flexor) muscle strength by 2·34 (*β* = –2·34, *P* = 0·004). For an increase in age of 1 year, gastrocnemius muscle strength increased by 3·58. Similarly, for children who were immunised, the gastrocnemius muscle strength increased by 3·53, and an increase in the birth weight of children (*β* = 2·43, *P* = 0·042) was positively associated with an increase in gastrocnemius muscle strength (Table 6).

## Discussion

This study aimed to investigate the association between muscle strength and MT among children aged 5–7 years old. The main findings of this study are (1) significantly lower (*P* < 0·001) grip, elbow flexor, quadriceps and gastrocnemius strength in children with MT compared with their WN peers, (2) regardless of the muscle group being tested, the nutritional status, sex and age are predictors for the amount of strength (Table 6) and (3) depending on the muscle group being tested other variables came in play to predict strength performance such as birth weight (grip and gastrocnemius strength), immunisation status (elbow flexor, quadriceps and gastrocnemius strength) and food insecurity (elbow flexor, quadriceps and gastrocnemius strength).

The differences in muscle strength in both upper and lower limbs found between the ***nutritional groups*** align with similar studies conducted in Italy (children 5–15 years), in Addis Ababa, Ethiopia (children 4–15 years)^(40,41)^, the United States (children 6–15 years)^(42)^, in South Africa and Ghana (children 5–12 years)^(43)^, in India^(21)^ and Argentina^(44)^ (children 6–10 years). The findings indicate that children with MT are at risk of different health consequences. A possible explanation for this difference could be that muscle is a major component of lean body mass, and acute malnutrition has a profound effect on the body mass as a whole and lean mass (fat-free mass), specifically as a response to starvation, causing gluconeogenesis. Hence, a deficit of lean body mass has a direct effect on muscle mass and strength. It has been shown that low muscle mass and strength contribute to an unfavorable metabolic profile in pediatric populations^(45,46)^. The odds of having an adverse level of any metabolic risk factors were associated with increased risk of chronic diseases, mortality and decreased hand grip strength^(47)^. Analyses of such grip strength and the strength of other muscles are primordial since they are indicators of nutritional status. Since muscle strength is a marker for physical fitness, which is crucial in performing daily activities, reduced strength may also impact a child’s overall functioning and well-being. For example, problems with grip strength may induce a decrease in fine motor skills, affecting the child’s performance at school in various ways, e.g. writing, which is required in all areas of learning^(45)^. Furthermore, reduced physical activity resulting from poor muscle strength may impact participation with peers. It can therefore induce diminished quality of life and further increase the risk of infection and mortality^(46)^.

It was also observed that strength increased with the ***chronological age*** of the children, which was a distinct difference by ***sex***. Our findings showed that muscle strength (torque) increased significantly with age for three muscle groups and the grip strength of the children. As a result, the mean muscle torque produced by 5-year-old children was significantly lower than the muscle strength produced by 6-year-old children, and the torque produced by 7-year-old subjects was higher than that of 5 and 6-year-old children. This is compatible with the study conducted in Michigan, USA^(34)^, and implies the coherent development of muscle tissue with chronological age. The effect of weight and sex in this finding was similar to the report of Ploegmakers *et al.* (2013)^(48)^. A possible reason for the significant positive result observed in males could be the greater muscle development commonly found in pre-adolescent males compared with females at the same growth stage because of increasing levels of circulating androgens in the males^(49)^.

Children living in ***poor households*** are at risk of undernutrition, which leads to low hand grip strength. Conversely, this study found that children living in food-secure households had low elbow flexor muscles and quadriceps muscle strength. Additionally, ***birth weight*** was a significant predictor of hand grip and gastrocnemius strength, which is consistent with the report of a study in Brazil^(50)^ that showed a significant positive correlation between birth weight and grip strength and motor performance of children. The findings have significant practical implications for the management of children with MT. In Ethiopia, management of moderately wasted children is implemented only in a few Integrated Management of Acute Malnutrition woredas, leaving children with MT in the other woredas without treatment, considering that the health extension programme addresses them. However, recently, there has been an effort to integrate the treatment of MT into routine health care. The findings imply the need for strengthening the speedy implementation of such efforts to reduce the consequences of MT on children.

Finally, the mean grip strength of 5–7 years children in this study was 5·65 (sd 2·04) and 4·15 (sd 2·56) for well-nourished and MT children, respectively, which is similar to the grip strength of Indian children of a similar age^(21)^, but slightly lower than children of the same age in the Netherlands^(48)^. The mean strength of the elbow flexor muscle in study participants was higher than children of a similar age from North America^(51)^. This may be due to the variation in body composition based on race, such that black children have less adipose tissue and greater muscle development than white children.

### Limitations and strengths of the study

The standardisation of muscle strength measurement and pretesting, and calculation of ICC, which showed excellent reliability, could be considered the study’s strengths. Muscle strength measurement was also performed in children aged 5–7 years, which is an advantageous age at which children start hand coordination and use their upper and lower extremity muscles for writing, throwing, jumping and others, and hence helps to apply effective strategies for improving muscle strength and promoting healthy lifestyles in early childhood. There is a limitation to this study that needs to be acknowledged. Social desirability bias could be a possibility, which is minimised by telling respondents that their response is meant for comparison and does not affect service use or privacy. In the age range of 5–7, some children may struggle to make maximal effort, which may have added noise to our data.

### Conclusion

The findings showed that children with MT had significantly lower muscle strength than their WN peers, indicating the negative effect of moderate wasting on muscle mass. Low muscle strength among MT children was also associated with maternal educational status, household food security, age of the child, sex of the child, birth weight and immunisation status of the child, implying the need for a multi-sectoral approach to alleviate low muscle strength associated with wasting. The results show the need for the inclusion of MT treatment into routine health care to reduce the consequences of MT on children. Future research on the development of normative data should preferably be collected across different regions in Ethiopia and a wider age range.
